# Supplementary material for: Global profiling of protein–DNA and protein–nucleosome binding affinities using quantitative mass spectrometry
Source: Nat Commun. 2018 Apr 25;9:1653. doi: 10.1038/s41467-018-04084-0 (PMC5916898; doi:10.1038/s41467-018-04084-0)
Supplement: Supplementary file 1 — Supplementary Information [file 41467_2018_4084_MOESM1_ESM.pdf]

Supplementary Information

Global profiling of protein-DNA and protein-nucleosome binding affinities using quantitative mass spectrometry

Makowski et al.

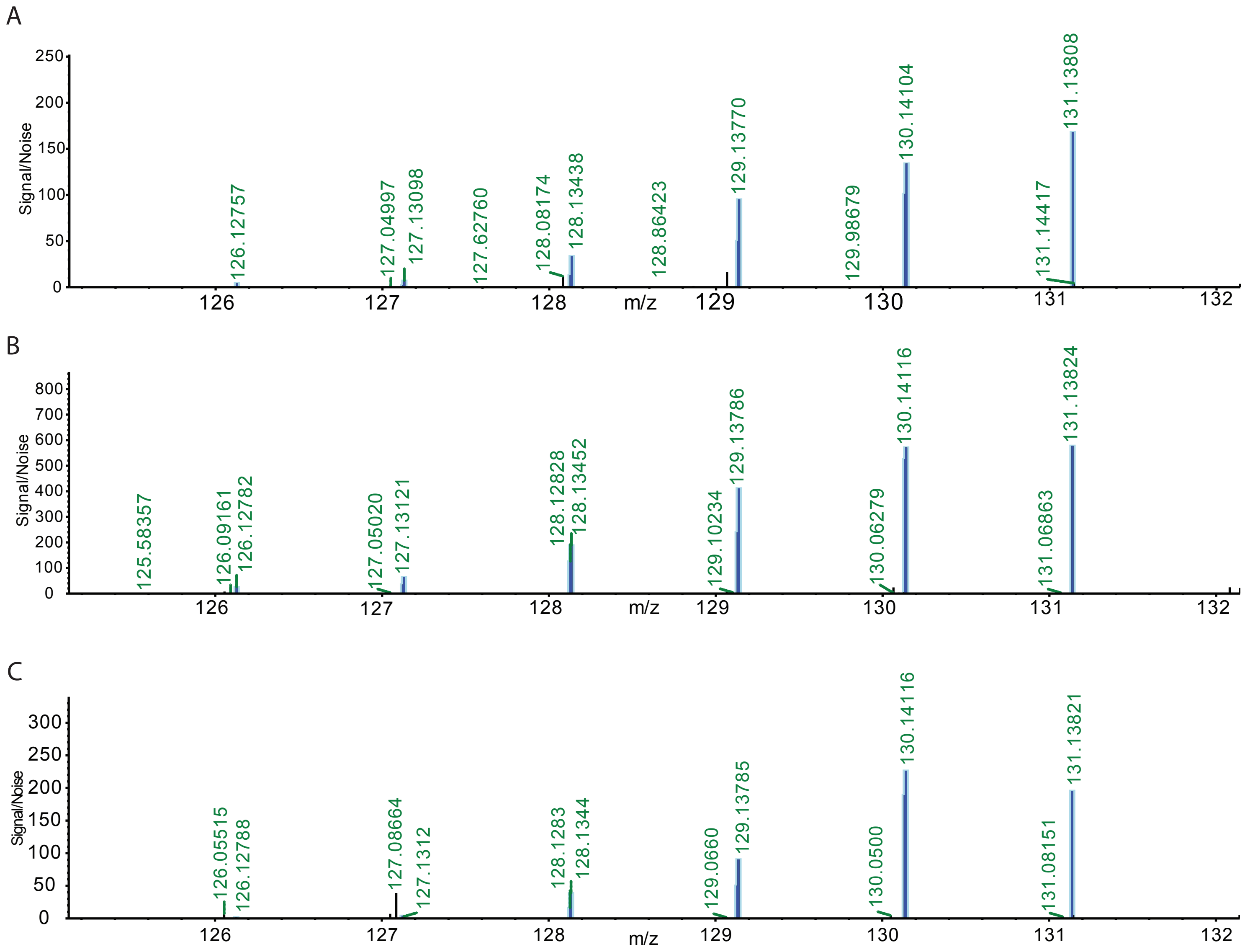

Supplementary Figure 1 Example MS3 TMT reporter ion spectra used for protein quantification

A) Example MS3 TMT reporter ion spectrum of an identified SP1 peptide. Only the low m/z range of the MS3 spectrum, where the TMT reporter ions are observed, is displayed for clarity. Plotted on the y-axis are signal-to-noise values from the orbitrap at 60,000 resolution.

B) Example MS3 TMT reporter ion spectrum of an identified SP3 peptide.

C) Example MS3 TMT reporter ion spectrum of an identified KLF4 peptide.

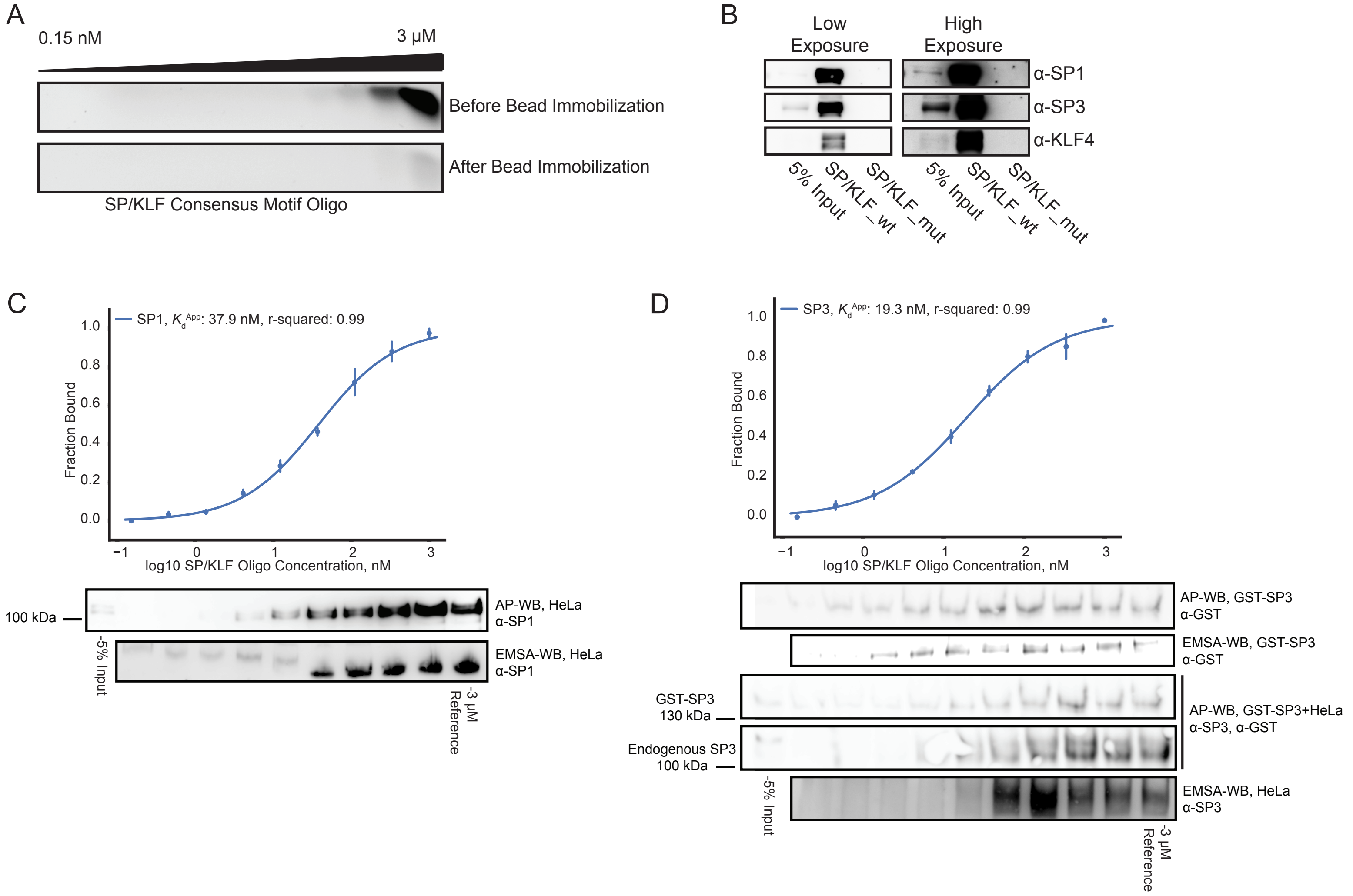

Supplementary Figure 2 Gel-based validation of oligo depletion and protein binding to SP/KLF motif

A) Agarose gel electrophoresis of SP/KLF consensus oligo titration, both before and after binding to streptavidin-sepharose beads, to indicate depletion of the oligo by bead immobilization.

B) Western blot analysis of canonical SP/KLF binding factors (SP1, SP3, and KLF4) binding to the SP/KLF wild-type oligonucleotide and not the SP/KLF mutated oligonucleotide.

C) Validation of mass spectrometry binding profile for SP1 to the SP/KLF consensus oligo. The mass spectrometry binding curve is shown above. Affinity purification western blot and electrophoretic mobility shift assay (EMSA) data are shown below. Mass spectrometry data points and protein bands are approximately aligned on the vertical axis.

D) Validation of mass spectrometry binding profile for SP3 to the SP/KLF consensus oligo. The mass spectrometry binding curve is shown above. Affinity purification western blot and electrophoretic mobility shift assay (EMSA) data of recombinant SP3 are shown below. Affinity purification western blot of recombinant SP3 spiked into HeLa lysate for pulldown analysis is shown next, with recombinant SP3 and endogenous SP3 separately noted. Finally, EMSA analysis of endogenous SP3 in HeLa nuclear lysate is shown at the bottom. Mass spectrometry data points and protein bands are approximately aligned on the vertical axis.

Binding curves were generated by fitting the parameters of the Hill equation including K_d^{App}. Each data point is the mean of three experiments (n=3), and the error bars represent the standard error of the mean.

Supplementary Figure 2

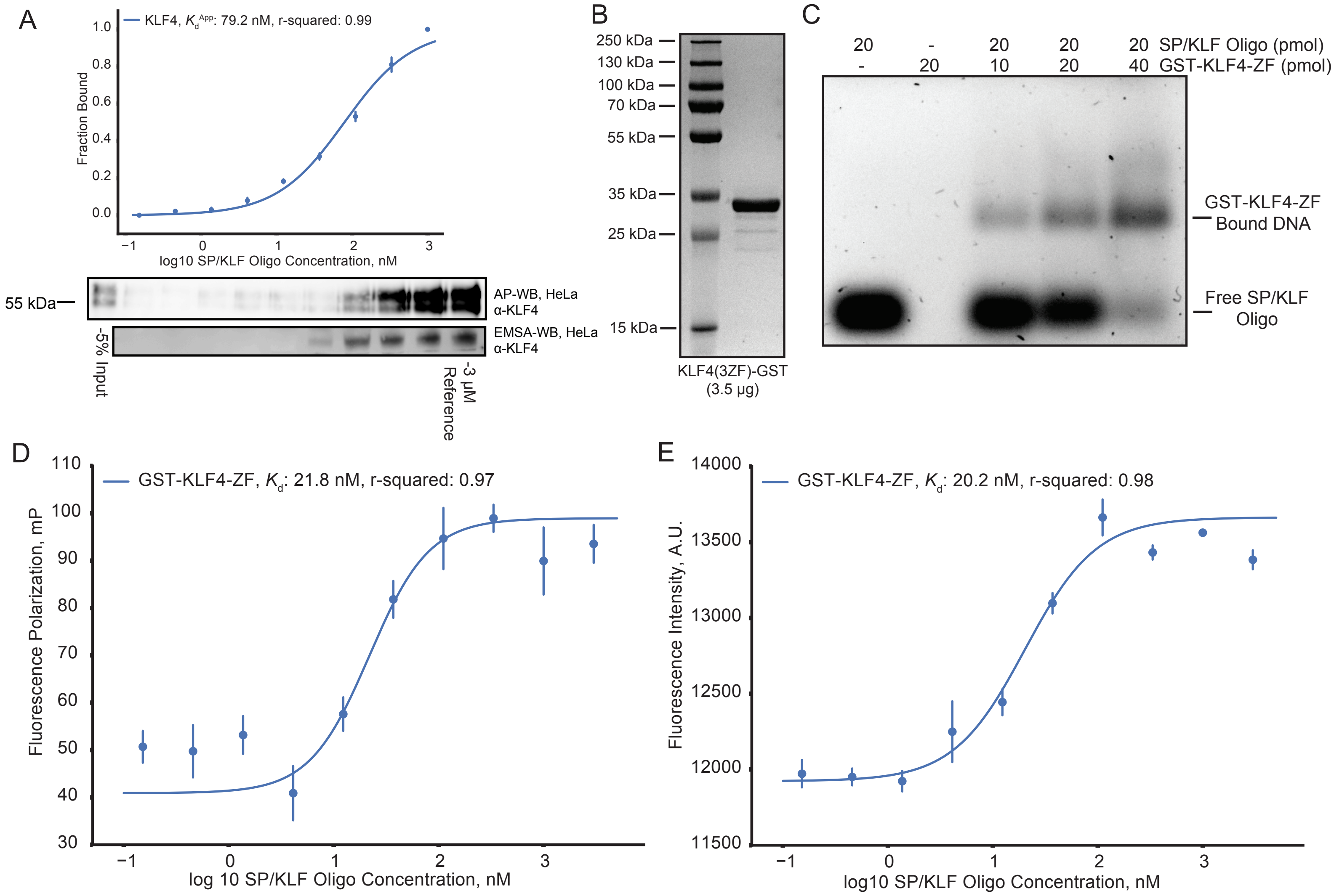

Supplementary Figure 3 KLF4-ZF fluorescence polarization and fluorescence intensity assays

A) Validation of mass spectrometry binding profile for KLF4 to the SP/KLF consensus oligo. The mass spectrometry binding curve is shown above. Affinity purification western blot and electrophoretic mobility shift assay (EMSA) data of endogenous KLF4 are shown below. Mass spectrometry data points and protein bands are approximately aligned on the vertical axis.

B) Imperial protein stain following SDS-PAGE of 3.5 µg of purified GST-KLF4-ZF recombinant protein.

C) Agarose gel electrophoretic mobility shift assay of GST-KLF4-ZF binding to the consensus SP/KLF oligonucleotide. Concentrations of either SP/KLF oligonucleotide or GST-KLF4-ZF are indicated above in picomoles.

D) Fluorescence polarization assay of GST-KLF4-ZF binding to the Cy5 labelled SP/KLF oligonucleotide.

E) Fluorescence intensity (de-quenching) assay of GST-KLF4-ZF binding to the Cy5 labelled SP/KLF oligonucleotide.

Binding curves were generated by fitting the parameters of the Hill equation including K_d^{App}. Each data point is the mean of three experiments (n=3), and the error bars represent the standard error of the mean.

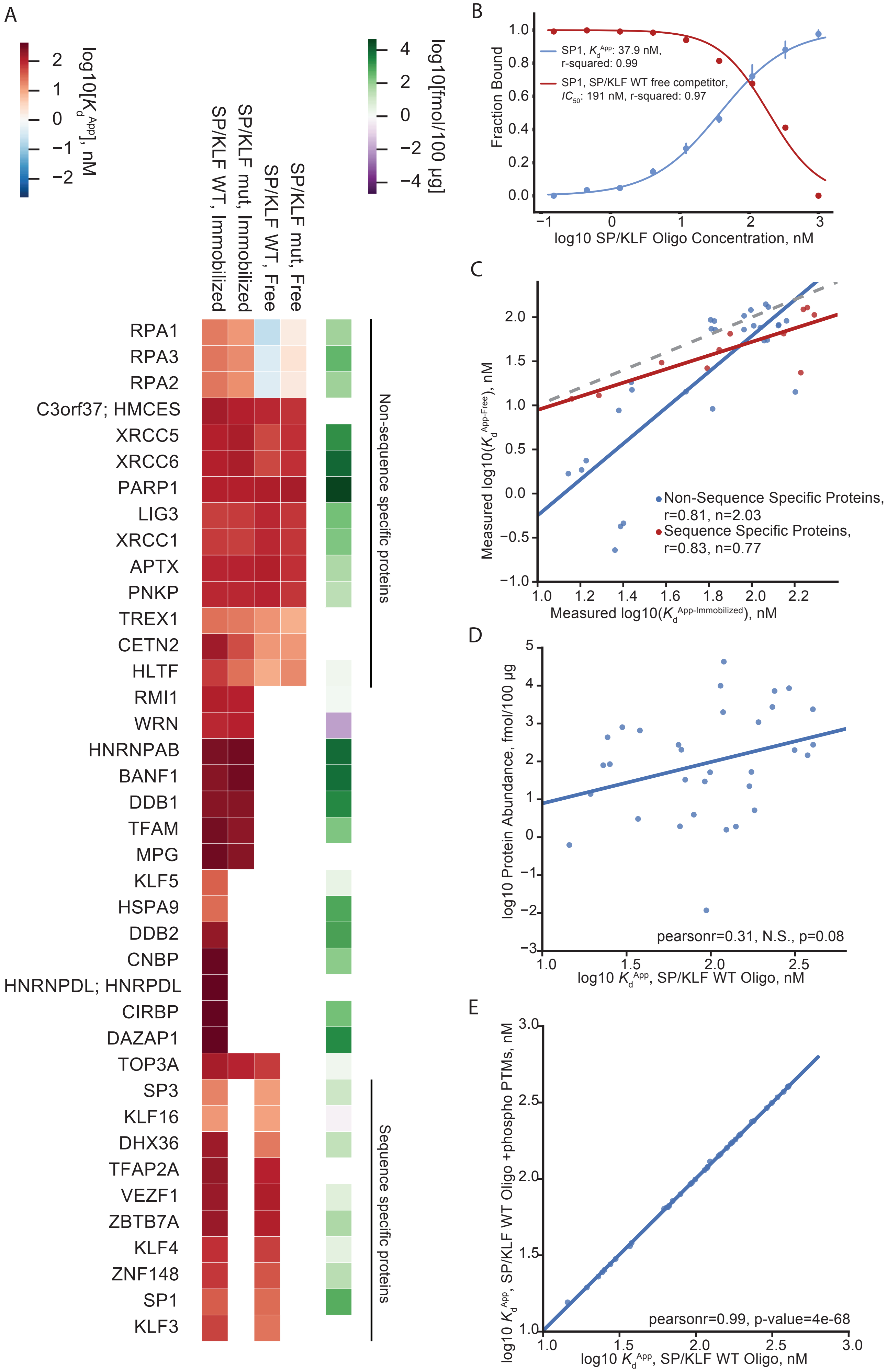

Supplementary Figure 4

Supplementary Figure 4 K_d^{App} calculated from competition experiment derived IC_{50} values correlates with K_d^{App} measured for immobilized baits for sequence-specific proteins

A) Heatmap analysis of log10-transformed K_d^{App} values for SP/KLF consensus oligonucleotide experiments, including with mutated sequence and with competition experiments. K_d^{App} values for competition experiments were calculated applying the Cheng-Prusoff correction to fit IC_{50} values as described in the Methods.

B) Hill-like curve for SP1 binding to the consensus SP/KLF motif (K_d^{App}) and for competition of SP1 by free SP/KLF motif (IC_{50}).

C) Regression analysis of K_d^{App} values measured in experiments with immobilized baits compared to KdApp values calculated from IC_{50} values measured in competition experiments. Sequence-specific proteins (as indicated in panel A based on specific high affinity binding for the wild-type SP/KLF oligonucleotide), are plotted in red, as is the correlation between experiments. Non-sequence specific proteins (as indicated in panel A based on high affinity binding for both the wild-type SP/KLF oligonucleotide and the mutated SP/KLF oligonucleotide), are plotted in blue, as is the correlation between experiments. r is the pearson correlation, and n is the slope of the regression line.

D) Regression analysis of K_d^{App} values measured for the wild-type SP/KLF oligonucleotide in immobilized bait experiments compared to absolute protein abundance in nuclear lysates. Each data point represents a single protein. The correlation is not significant at a p-value of 0.05.

E) Regression analysis of K_d^{App} values measured for the wild-type SP/KLF oligonucleotide in immobilized bait experiments. Two analysis were performed and plotted, as described in the methods, either considering or not considering STY peptide phosphorylation. Each data point represents a single protein.

Binding curves were generated by fitting the parameters of the Hill equation including K_d^{App}. Each data point is the mean of three experiments (n=3), and the error bars represent the standard error of the mean.

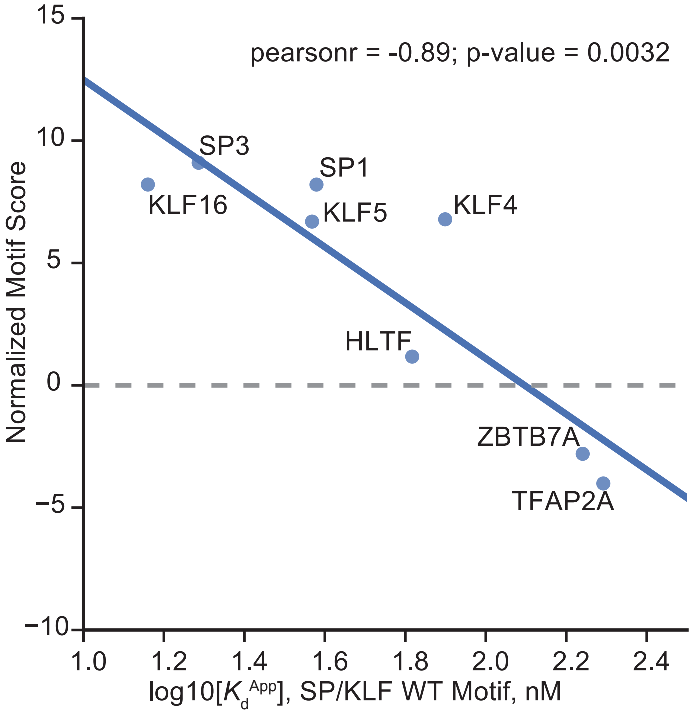

Supplementary Figure 5 Observed K_d^{App} correlates with transcription factor motif score
Regression analysis between the log10-transformed K_d^{App} value for sequence-specific SP/KLF wild-type oligonucleotide binding factors and the normalized JASPAR motif score for the SP/KLF consensus oligo as defined in the Methods. The grey dashed line, therefore, indicates the significance threshold for each individual factor.

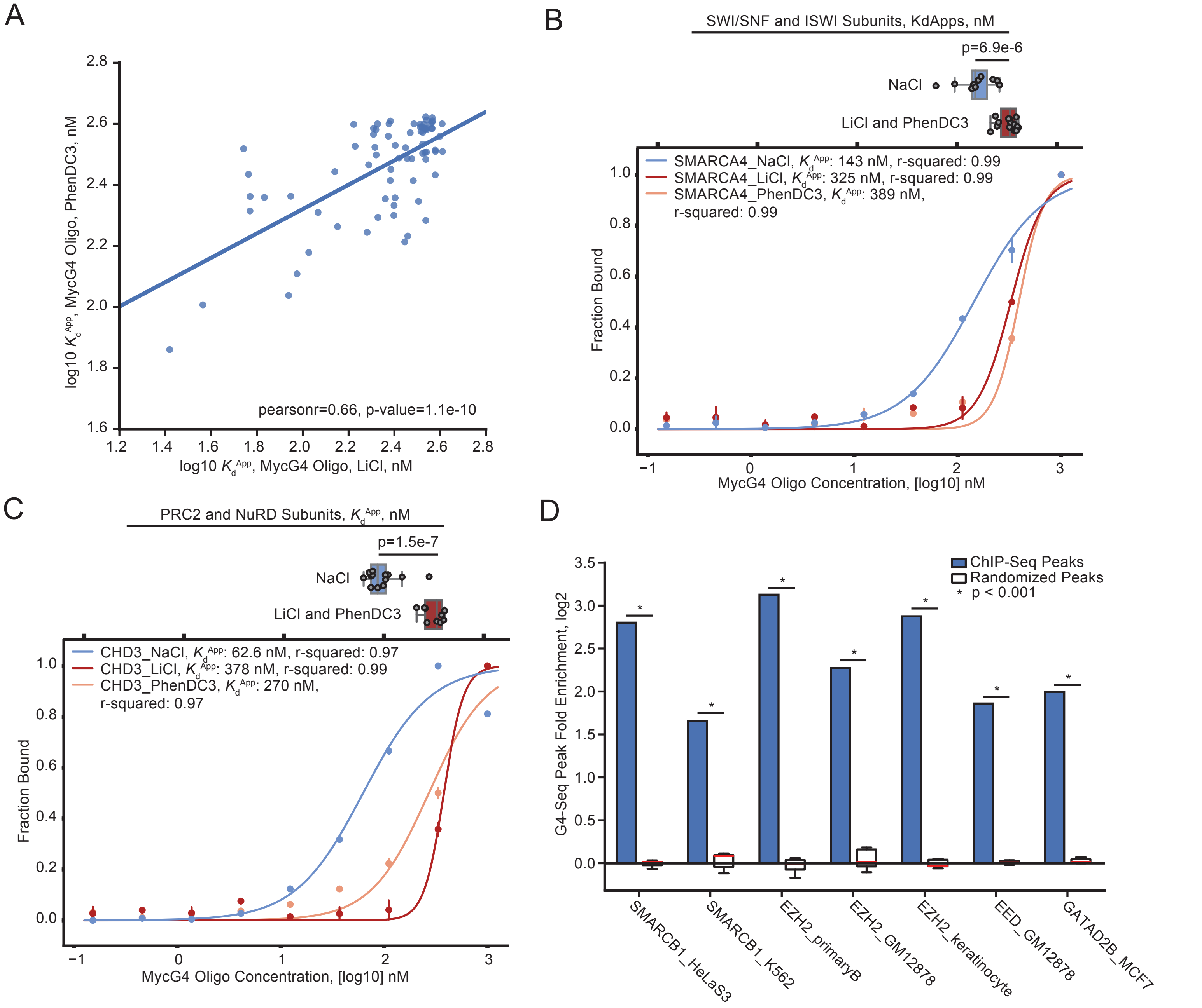

Supplementary Figure 6 Chromatin modifying complexes bind with lower K_d^{App} to the mycG4 sequence in G4-permissive conditions

A) Regression analysis of K_d^{App} values measured for the mycG4 ssDNA oligonucleotide in LiCl binding conditions compared to PhenDC3 binding conditions as described in the methods. Each data point represents a single protein.

B) Binding curves for SMARCA4 (Fig. 2A, Cluster 7), a SWI/SNF catalytic subunit, are shown for the mycG4 oligonucleotide in NaCl (G4-permissive), LiCl (G4 non-permissive), and PhenDC3 (G4 ligand) binding conditions. Above are boxplots of all identified KdApp values SWI/SNF and ISWI subunits in these conditions, with LiCl and PhenDC3 grouped together given their similar overall effect (also shown in panel A).

C) Binding curves for CHD3 (Cluster 7), a NuRD catalytic subunit, are shown for the mycG4 oligonucleotide in NaCl (G4-permissive), LiCl (G4 non-permissive), and PhenDC3 (G4 ligand) binding conditions. Boxplots and data points are colored as in panel B.

D) Permutation based testing of G4-seq (generated in primary B cells) peak enrichment in ENCODE ChIP-seq peaks for SWI/SNF, PRC2, and NuRD subunits in various cell lines. ChIP-seq peaks were randomized 1000 times across the genome, and the distribution of enrichment values for randomized G4-seq intersected peak counts, compared to the mean of peak randomizations, is displayed as a white boxplot with parameters as described previously. The enrichment of ChIP-seq peak enrichment compared to peak randomizations is indicated as a blue bar. An empirical p-value was calculated by comparing the distribution of randomized peak intersections with the true peak intersections.

Boxplots are displayed such that the center line is the median of the distribution, the box represents the first and third quartile of the data, and the whiskers represent 1.5 inter-quartile ranges. Significance is indicated based on a two-sided t-test, with all measured values per sample, as indicated by the colored grouping, treated as sample populations.
Binding curves were generated by fitting the parameters of the Hill equation including K_d^{App}. Each data point is the mean of two experiments (n=2), and the error bars represent the standard error of the mean.

A

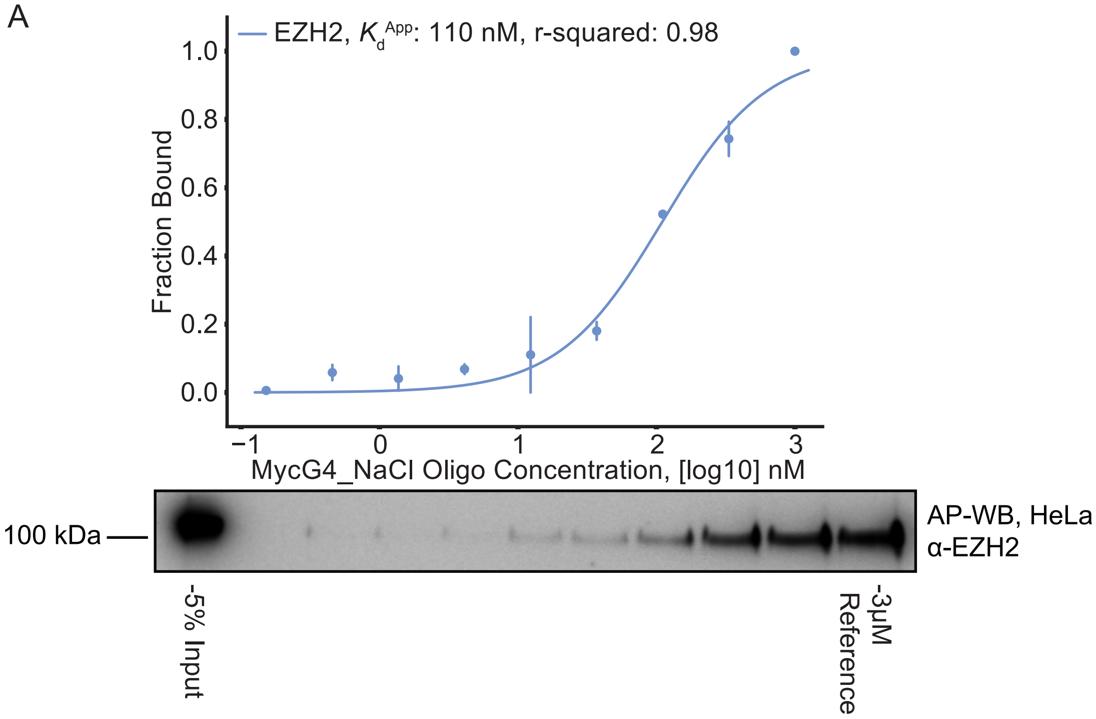

B

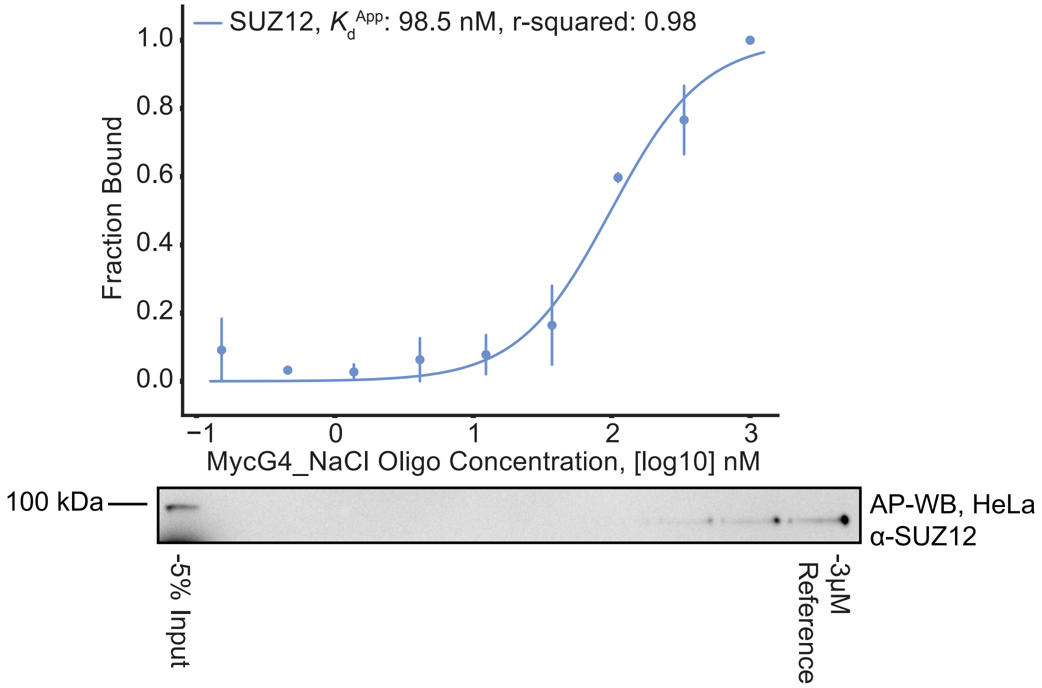

C

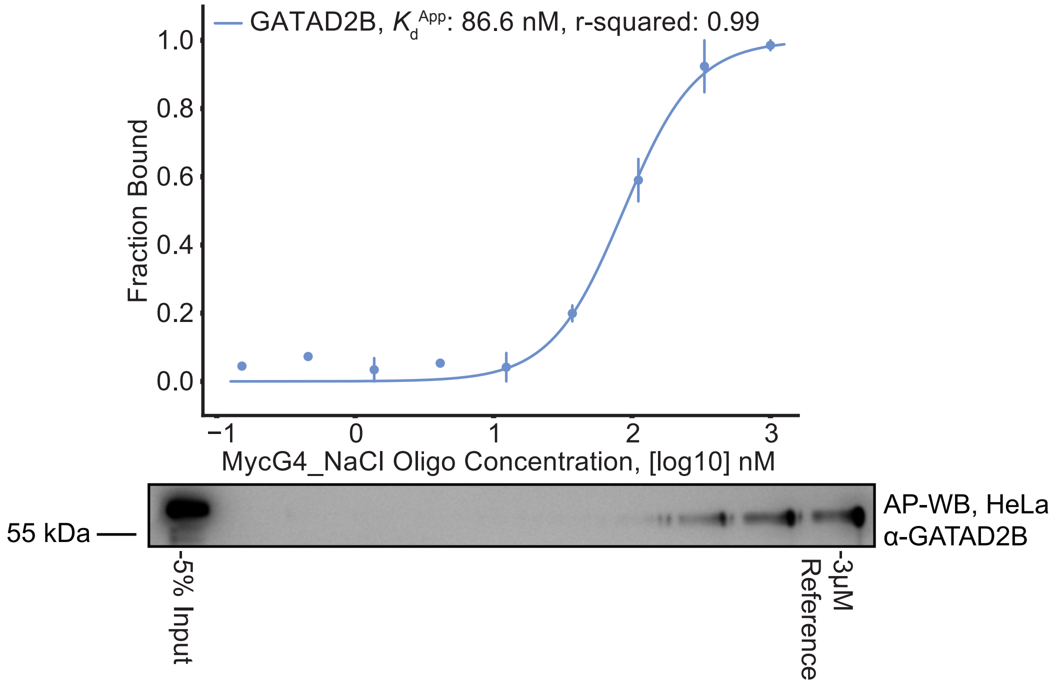

Supplementary Figure 7

Supplementary Figure 7 Western blot validation of PRC2 and NuRD binding to the mycG4 sequence

A) Validation of mass spectrometry binding profiles for EZH2 (PRC2 subunit, Fig. 2A, Cluster 7) to the mycG4 consensus oligo. The mass spectrometry binding curve is shown above with affinity purification western blot data shown below. Mass spectrometry data points and protein bands are approximately aligned on the vertical axis.

B) Validation of mass spectrometry binding profiles for SUZ12 (PRC2 subunit, Cluster 7) to the mycG4 consensus oligo. The mass spectrometry binding curve is shown above, with affinity purification western blot data shown below. Mass spectrometry data points and protein bands are approximately aligned on the vertical axis.

C) Validation of mass spectrometry binding profiles for GATAD2B (NuRD subunit, Cluster 7) to the mycG4 consensus oligo. The mass spectrometry binding curves are shown above, with affinity purification western blot data shown below. Mass spectrometry data points and protein bands are approximately aligned on the vertical axis.

Binding curves were generated by fitting the parameters of the Hill equation including K_d^{App}. Each data point is the mean of two experiments (n=2), and the error bars represent the standard error of the mean.

Supplementary Figure 7

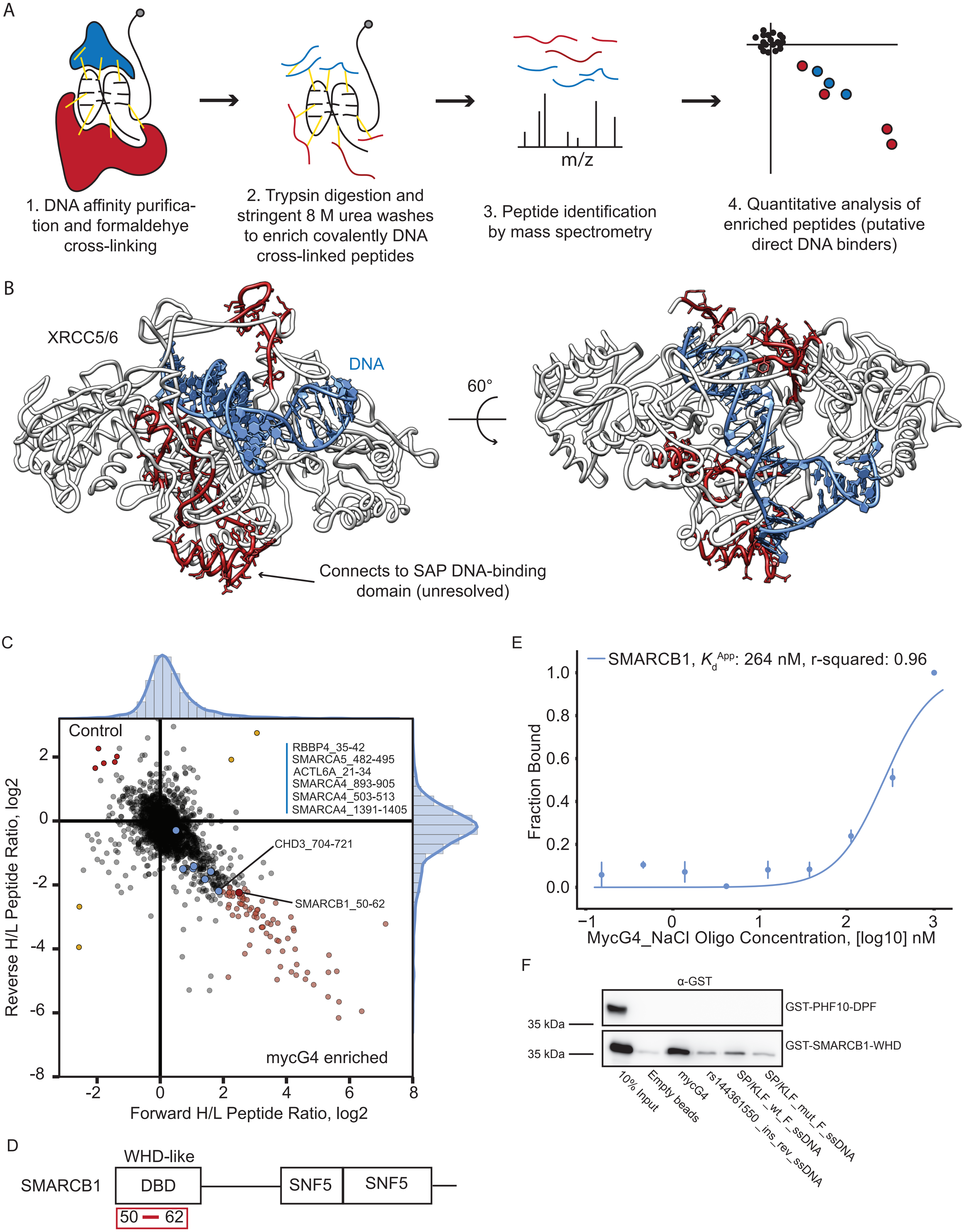

Supplementary Figure 8

Supplementary Figure 8 The SMARCB1 winged helix domain directly binds to and prefers the mycG4 sequence

A) Workflow for formaldehyde protein-DNA cross-linking experiments. Briefly, proteins were covalently cross-linked to DNA oligonucleotides. Proteins were digested and non-linked peptides were removed by stringent 8 M urea washes. After de-crosslinking, enriched peptides were identified by tandem mass spectrometry analysis, and enrichment was quantified using dimethyl chemical labeling.

B) Significantly enriched peptides for the XRCC5/6 heterodimer are colored on the crystal structure (PDB:1JEY) in red. Co-crystallized DNA is colored in blue.

C) Outlier plot of enriched peptides for the mycG4 sequence. Each axis is the log2 transformed light/heavy dimethyl ratio from a single replicate. Each data point is a peptide. Enriched peptides using a significance cutoff of 1.5 interquartile ranges are colored in red. Background proteins are colored in black. Background proteins that are members of the SWI/SNF, PRC2, or NuRD complexes are colored in blue.

D) Domain schematic of SMARCB1. The enriched peptide identified as significant in panel C is colored in red below the protein cartoon. Amino acid peptide indices are indicated by number.

E) Binding curve for SMARCB1 (Fig. 2A, Cluster 8) to the mycG4 ssDNA oligo in NaCl binding conditions. The binding curve were generated by fitting the parameters of the Hill equation including K_d^{App}. Each data point is the mean of three experiments (n=3), and the error bars represent the standard error of the mean.

F) DNA pulldown and western blot experiments with bacterial lysates expressing either the SMARCB1 winged helix domain or the PHF10 double PHD finger. DNA pulldowns were performed as described as in the Methods using the mycG4 sequence and a variety of control sequences and analyzed by western blot. rs144361550 is a oligonucleotide representing a small insertion/deletion single nucleotide polymorphism predicted by computation to be G4-forming but characterized biochemically as non-G4 forming. The SP/KLF wild-type ssDNA is a GC-rich control sequence which is not predicted to form G4 structures. The SP/KLF mutated ssDNA is an AT-rich control sequence which is similarly not predicted to form G4 structures.

Supplementary Figure 8

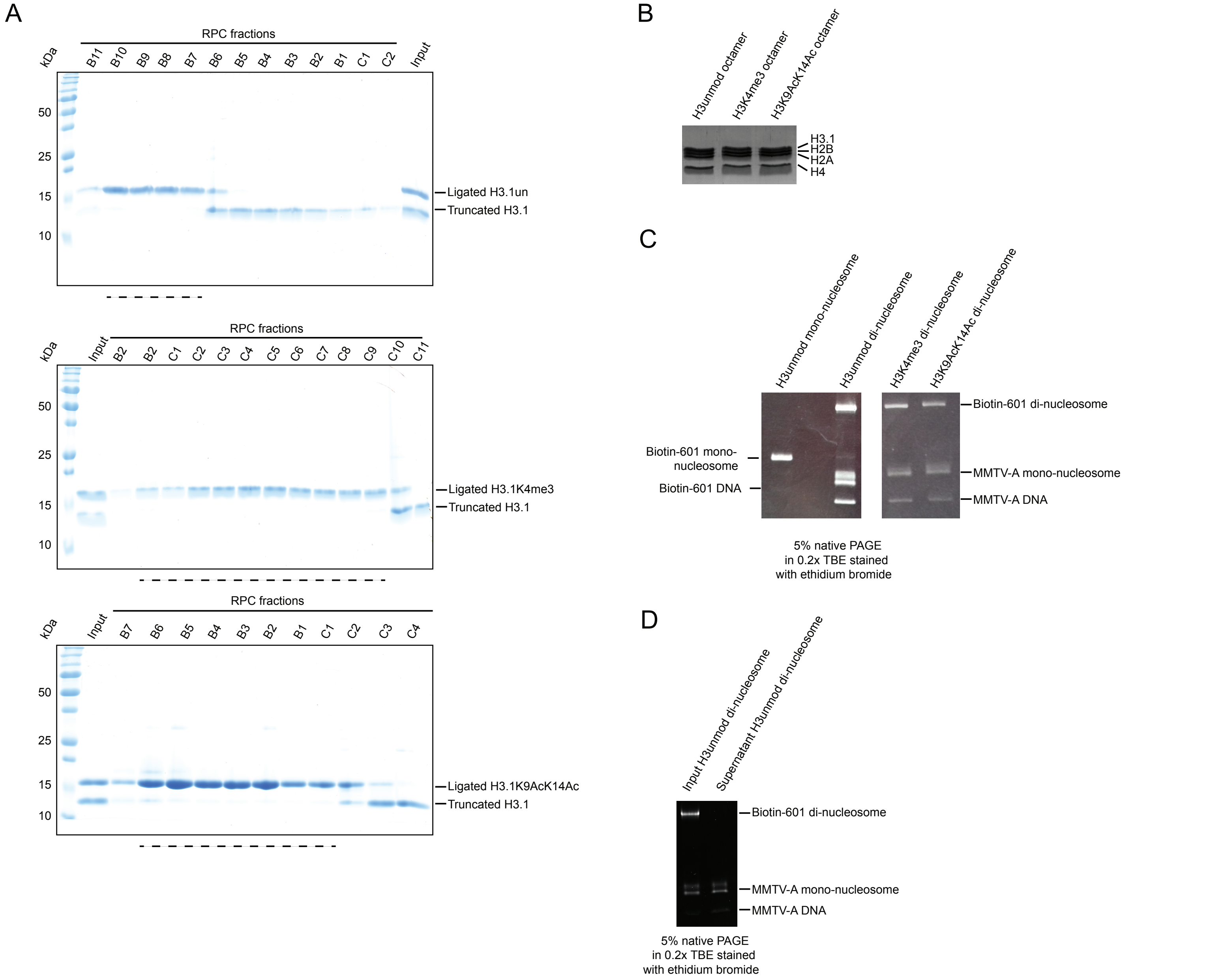

Supplementary Figure 9 Quality control checks of mono-nucleosomes and modified and unmodified di-nucleosomes

A) 17.5% SDS-PAGE gels stained with Coomassie Brilliant Blue of Reverse-phase chromatography fractions indicating the separation of ligated full-length histone H3 (unmodified, K4me3 and K9AcK14Ac) and the truncated histone H3.1. Fractions underlined with a dashed line were pooled for refolding into histone octamers.

B) 17.5% SDS-PAGE gels stained with Coomassie Brilliant Blue of the H3unmodified, H3K4me3 and H3K9AcK14Ac histone octamers.

C) 5% native PAGE gels stained with ethidium bromide or SYBR safe of the biotinylated mono-/di-nucleosomes. The free DNA and band-shifted nucleosomes are indicated.

D) Input di-nucleosome substrate and the supernatant after binding to streptavidin beads indicating that the free competitor MMTV-A DNA and mono-nucleosomes do not bind to the beads and the biotinylated di-nucleosomes do bind.

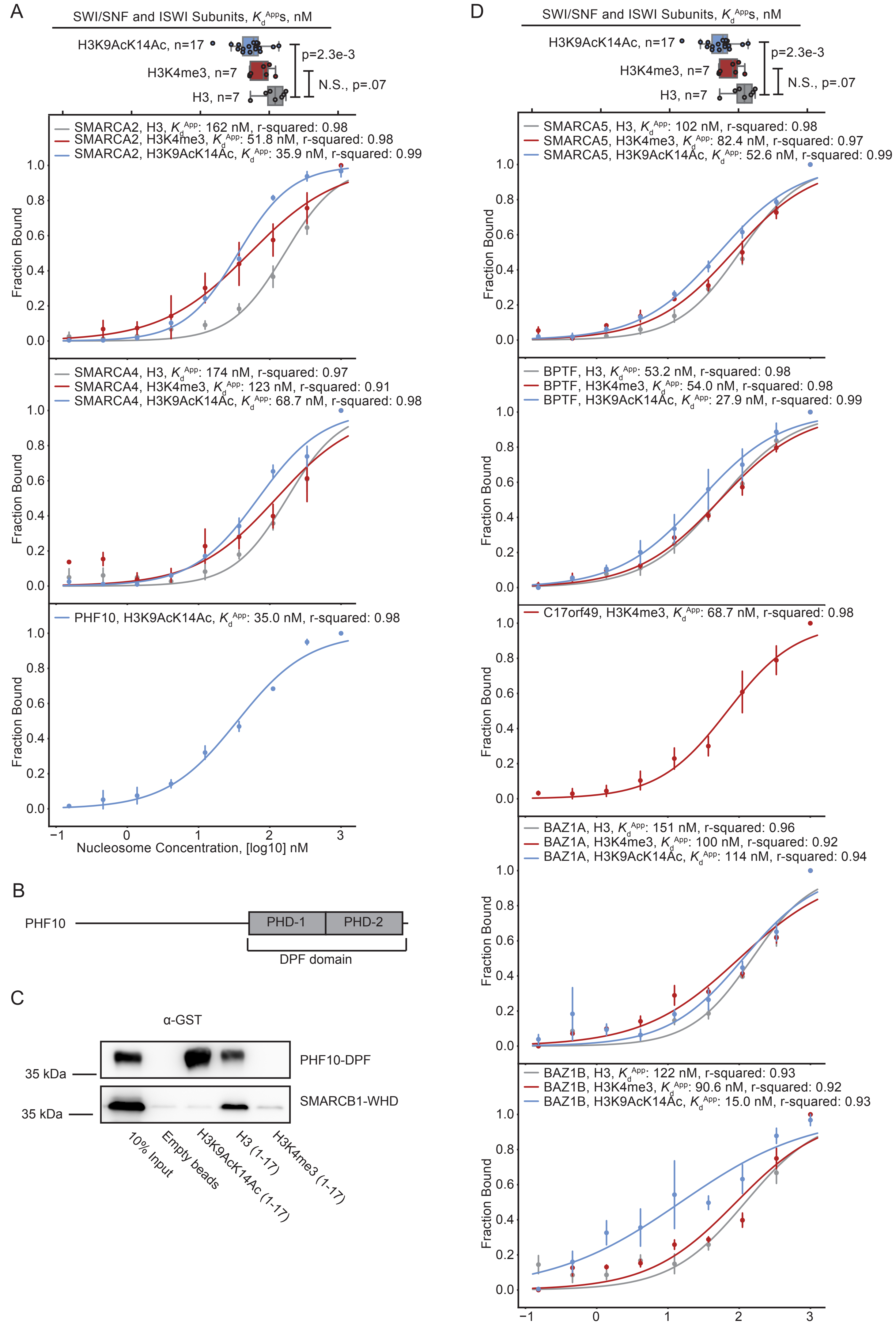

Supplementary Figure 10

Supplementary Figure 10 SWI/SNF and ISWI di-nucleosome binding affinity is modulated by H3 modifications
A) Binding curves for SMARCA2 (Fig. 3B, Cluster 5) and SMARCA4 (Cluster 5), SWI/SNF catalytic subunits, and PHF10 (Cluster 3), a SWI/SNF accessory subunit, are shown for unmodified, H3K4me3, and H3K9AcK14Ac modified di-nucleosomes. Above are boxplots of all identified K_d^{App} values SWI/SNF and ISWI subunits for these substrates.
B) Domain schematic of PHF10. The double PHD finger (DPF) domain used for cloning, protein expression, and histone peptide pulldown experiments is specifically indicated.
C) Histone peptide pulldown and western blot experiments with bacterial lysates expressing either the PHF10 double PHD finger or the SMARCB1 winged helix. Peptide pulldowns were performed as described as in the Methods using the H3 peptides with the indicated post-translational modifications.
D) Binding curves for SMARCA5 (Cluster 3), ISWI catalytic subunit, and BPTF (Cluster 3), C17orf49 (Cluster 1), BAZ1A (Cluster 4), and BAZ1B (Cluster 3), SWI/SNF accessory subunits, are shown for unmodified, H3K4me3, and H3K9AcK14Ac modified di-nucleosomes. Above are boxplots of all identified K_d^{App} values SWI/SNF and ISWI subunits for these substrates. Significance is indicated based on a two-sided t-test, with all measured values per sample, as indicated by the colored grouping, treated as sample populations.

Boxplots are displayed such that the center line is the median of the distribution, the box represents the first and third quartile of the data, and the whiskers represent 1.5 inter-quartile ranges. Significance is indicated based on a two-sided t-test, with all measured values per sample, as indicated by the colored grouping, treated as sample populations.
Binding curves were generated by fitting the parameters of the Hill equation including K_d^{App}. Each data point is the mean of three experiments (n=3), and the error bars represent the standard error of the mean.

Supplementary Figure 2a

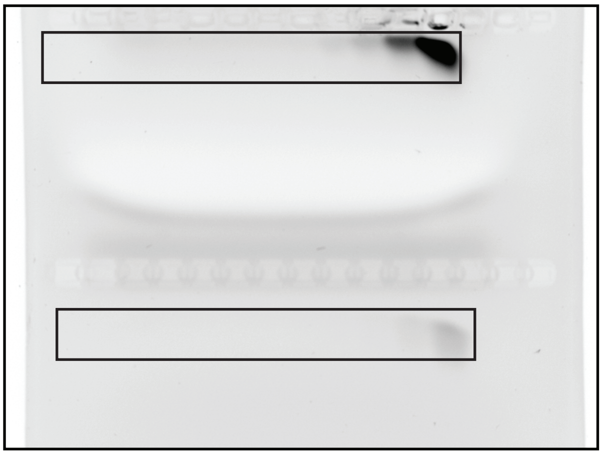

Supplementary Figure 2b

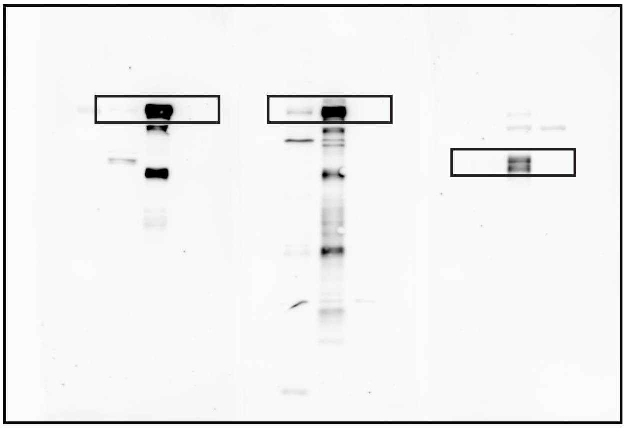

Supplementary Figure 2c

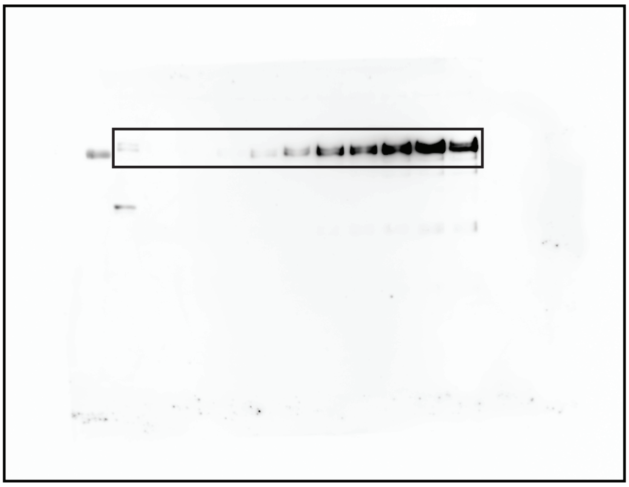

Supplementary Figure 2c

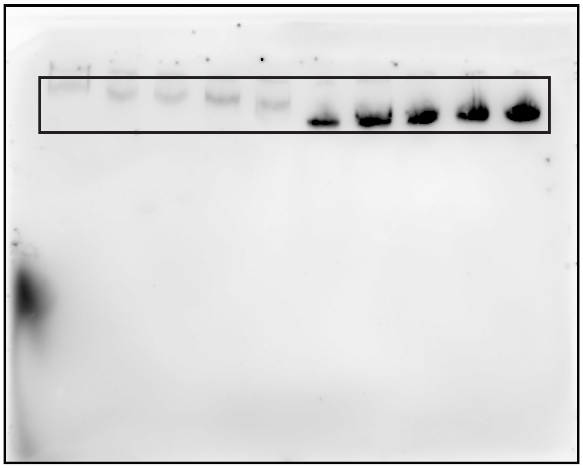

Supplementary Figure 2d

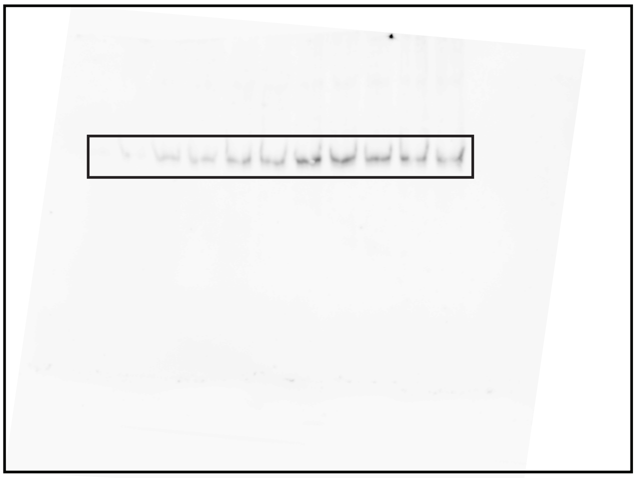

Supplementary Figure 2d

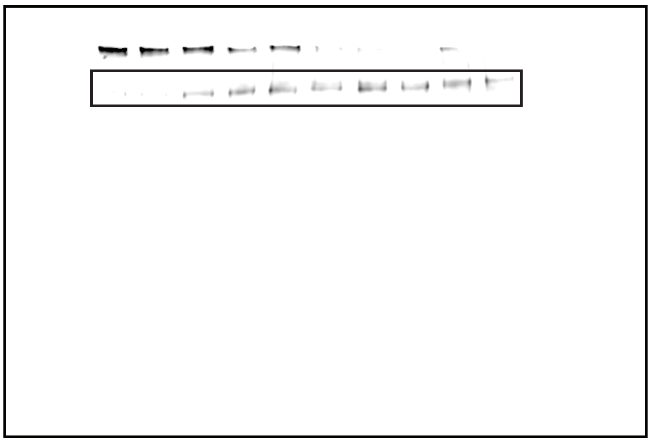

Supplementary Figure 2d

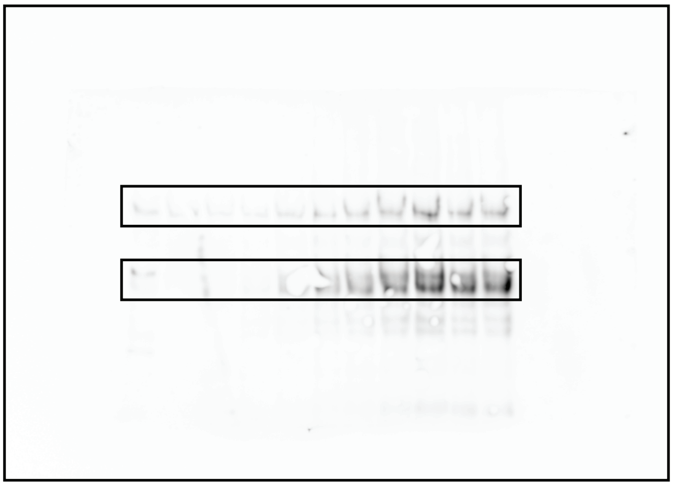

Supplementary Figure 2d

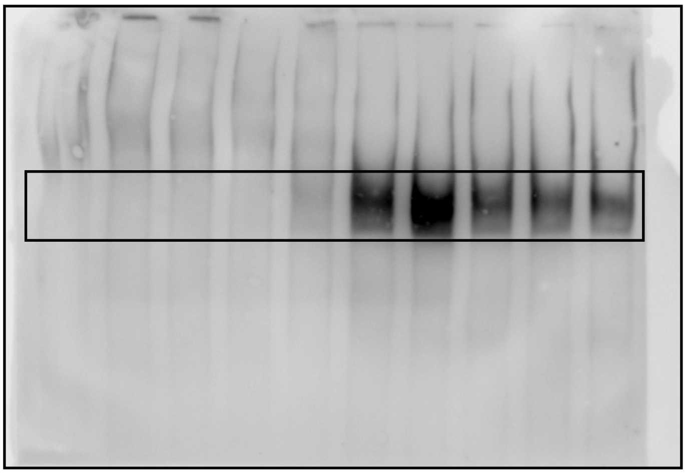

Supplementary Figure 3a

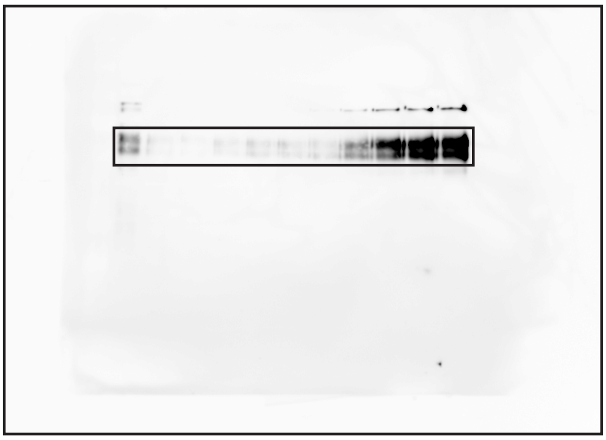

Supplementary Figure 3a

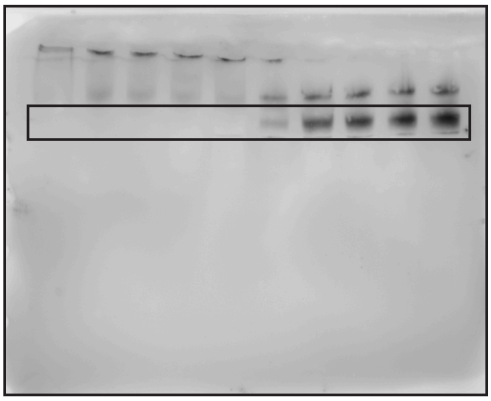

Supplementary Figure 3b

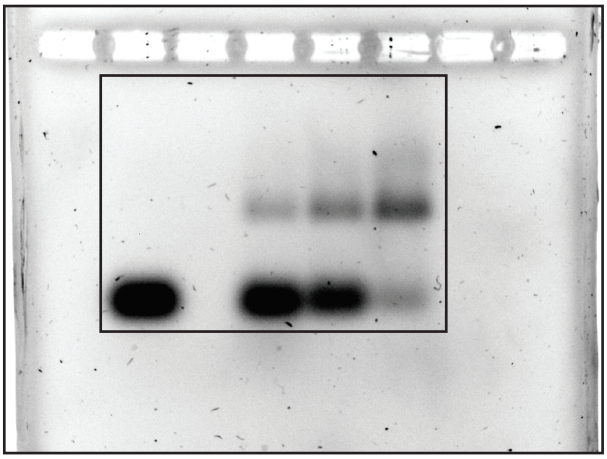

Supplementary Figure 7a

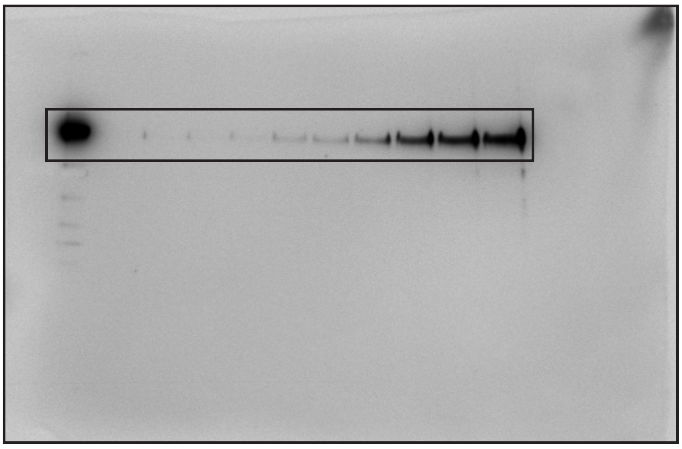

Supplementary Figure 7b

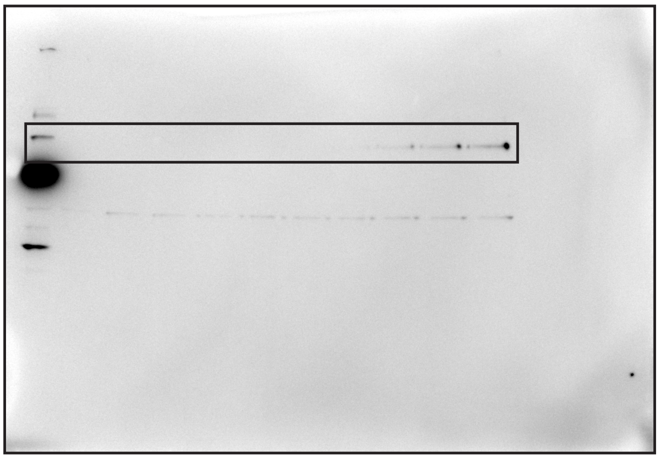

Supplementary Figure 7c

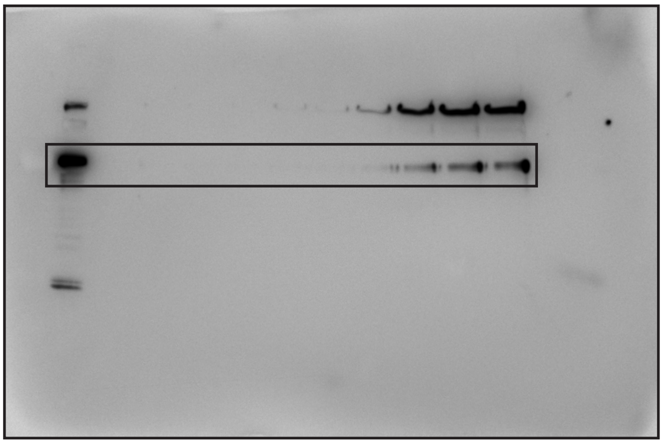

Supplementary Figure 8f

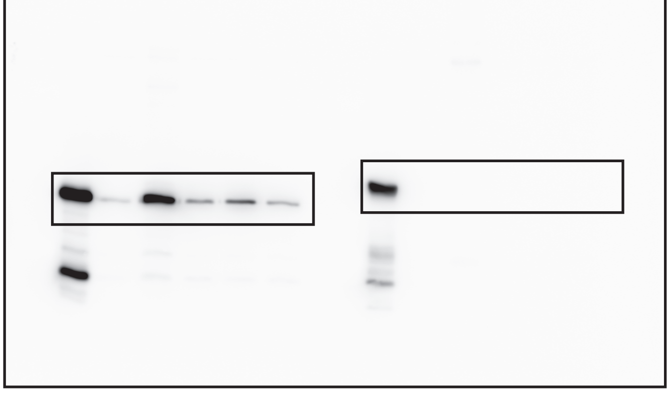

Supplementary Figure 10c

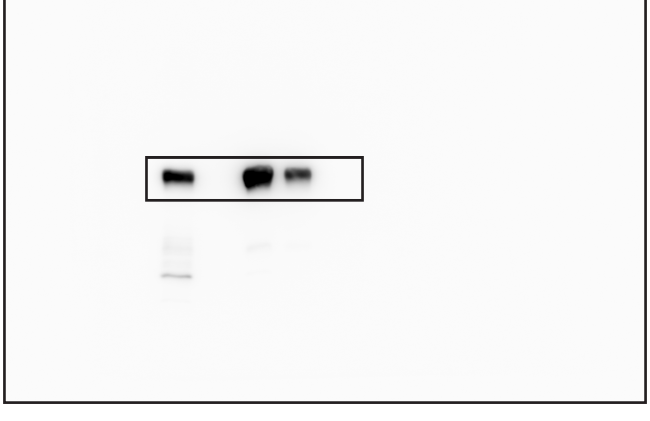

Supplementary Figure 10c

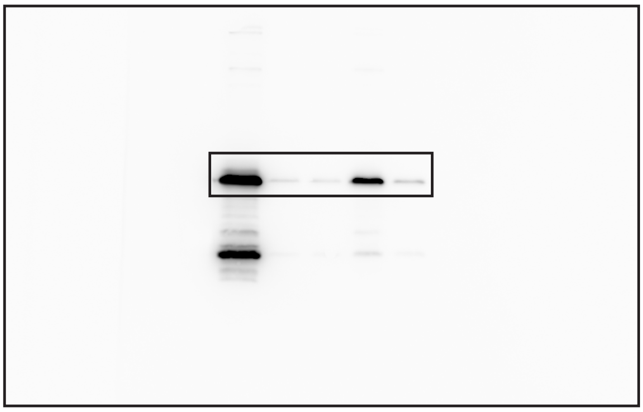

Supplementary Figure 11 Raw blot images for gel electrophoresis analysis shown in this study
The figure each raw image is related to is indicated in text above each blot. Cropped lanes are indicated by black box.

Name	Sequence	Sequence 2
Sp/KLF Wild-type Consensus	/5Biosg/GAGAGCCCCGCCCCCTGGCT	AGCCAGGGGGCGGGGCTCTC
Sp/KLF Mutated	/5Biosg/GAGAGAAAATAAAACTGGCT	AGCCAGTTTTATTTTCTCTC
AP-1	/5Biosg/AGTCGGCTAGCTGACTCAGGATGTCC	GGACATCCTGAGTCAGCTAGCCGACT
CTCF	/5Biosg/TCAGAGTGGCGGCCAGCAGGGGGCGCCCTTGCCAGA	TCTGGCAAGGGCGCCCCCTGCTGGCCGCCACTCTGA
E-box	/5Biosg/GGAAGCAGACCACGTGGTCTGCTTCC	GGAAGCAGACCACGTGGTCTGCTTCC
NF-Y	/5Biosg/ATTGACCAATCAGAGGTAGGATGAT	ATCATCCTACCTCTGATTGGTCAAT
TATA-box	/5Biosg/GCGGCGCTCTATATAAGTGGGCAGTG	CACTGCCCACTTATATAGAGCGCCGC
TEAD	/5Biosg/TCGGGACCCAGGCCTGGAATGTTTCCACC	GGTGGAAACATTCCAGGCCTGGGTCCCGA
MycG4	/5Biosg/TGGGGAGGGTGGGGAGGGTGGGGAAGG	
Telomere	/5Biosg/TTAGGGTTAGGGTTAGGGTTAGGG	
rs144361550_PARP1_ins_biotin_rev (predicted G4, biochemically characterized as non-G4)	/5Biosg/GAGCGAGCGGGCCCGGGCCCgggcccTCGGAGCGGCACTTGGGGCC	

Supplementary Table 1 Oligonucleotide baits used in this study.
Oligonucleotides were ordered as described above from IDT. IDT notation for oligonucleotide modifications is included in the sequence. Biotin free sequences for competition experiments were ordered without biotinylation. dsDNA oligonucleotides were annealed before use as described in the methods.

Antibodies			
**Factor**	**Source**	**Catalog Number**	**Ab Dilution Used**
SP1	Sigma	S9809	1:100
SP3	abcam	ab72594	1:500
KLF4	Sigma	HPA002926	1:1000
EZH2	Cell signaling technologies	5246	1:500
GATAD2B	Bethyl laboratories	A301-282A	1:2000
Suz12	abcam	ab12073	1:2000
GST	Thermo	MA4-004	1:1000
**Recombinant proteins**			
**Protein**	**Source**	**Additional Information**	
SP3	Abnova	Catalog Number: H00006670-P01	
KLF4-ZF	Spruijt et al. Cell. 2012.		
PHF10 Double PHD finger domain (aa358-aa498)	This study	For primer: CCCGGATCCCCAAACGTTCTGTACTGTCC	Rev Primer: GGGAAGCTTATTATCCCTCTTTGCTGTTTTTC
SMARCB1 winged helix domain (aa1-aa112)	This study	For primer: CAATCCATGGGAATGATGATGATGGCGCTGAG	Rev Primer: TCGGGATCCTTATTAGATGGACACAGCCTTGTAC

Supplementary Table 2 Antibodies and recombinant proteins used in this study.

Factor	Cell Line	Database	Accession Number
**ChIP-Sequencing**			
SMARCB1	HeLaS3	ENCODE	ENCFF002CSN
SMARCB2	K562	ENCODE	ENCFF993YKH
GATAD2B	MCF7	ENCODE	ENCFF046BRP
EED	GM12878	ENCODE	ENCFF023ALY
EZH2	NHEK Keratinocytes	ENCODE	ENCFF002CFB
EZH2	GM12878	ENCODE	ENCFF615NYO
EZH2	Primary human CD20+ B-cells, RO01794	ENCODE	ENCFF434OEY
**G4-sequencing**			
Induced G4 (K+, PDS), plus strand	Primary human B-cells, NA18507	GEO	GSE63874
Induced G4 (K+, PDS), minus strand	Primary human B-cells, NA18507	GEO	GSE63874

Supplementary Table 3 Publically available sequencing data sets used in this study.
