# Supplementary material for: Global profiling of protein–DNA and protein–nucleosome binding affinities using quantitative mass spectrometry
Source: Nat Commun. 2018 Apr 25;9:1653. doi: 10.1038/s41467-018-04084-0 (PMC5916898; doi:10.1038/s41467-018-04084-0)
Supplement: Supplementary file 3 — Description of Additional Supplementary Files [file 41467_2018_4084_MOESM3_ESM.pdf]

Description of Additional Supplementary Files

File Name: Supplementary Data 1

Description: K_d^{App} values measured in this study. Sheet 1 shows K_d^{App} values measured for SP/KLF consensus motif experiments including IC_{50} values from competition experiments. Sheet 2 shows K_d^{App} values measured for motif survey experiments including with the mycG4 sequence. Sheet 3 shows K_d^{App} values measured in nucleosome and modified nucleosome experiments. All values are in units of nM.

File Name: Supplementary Data 2

Description: Absolute protein quantification of HeLa nuclear lysate
